# Mid- to Long-Term Magnetic Resonance Imaging Results of Two Prolapse Surgeries for Apical Defect: A Secondary Analysis of a Randomized Controlled Trial

**DOI:** 10.1055/s-0040-1718441

**Published:** 2021-01-29

**Authors:** Luiz Carlos Santos Junior, Luiz Gustavo Oliveira Brito, Edilson Benedito de Castro, Sergio Dertkigil, Cassia Raquel Teatin Juliato

**Affiliations:** 1 Department of Obstetrics and Gynecology, Pelvic Floor Dysfunction Division, Faculdade de Ciências Médicas, Universidade Estadual de Campinas, Campinas, SP, Brazil

**Keywords:** pelvic organ prolapse, magnetic resonance imaging, cross-sectional studies, prolapso dos órgãos pélvicos, ressonância magnética, estudos transversais

## Abstract

**Objective** Magnetic resonance imaging (MRI) has been considered another tool for use during the pre- and postoperative periods of the management of pelvic-organ prolapse (POP). However, there is little consensus regarding its practical use for POP and the association between MRI lines of reference and physical examination. We aimed to evaluate the mid- to long-term results of two surgical techniques for apical prolapse.

**Methods** In total, 40 women with apical POP randomized from 2014 to 2016 underwent abdominal sacrocolpopexy (ASC group; n = 20) or bilateral vaginal sacrospinous fixation with an anterior mesh (VSF-AM group; n = 20). A physical examination using the POP Quantification System (POP-Q) for staging (objective cure) and the International Consultation on Incontinence Questionnaire-Vaginal Symptoms (ICIQ-VS: subjective cure), were applied and analyzed before and one year after surgery respectively. All MRI variables (pubococcigeous line [PCL], bladder base [BB], anorectal junction [ARJ], and the estimated levator ani subtended volume [eLASV]) were investigated one year after surgery. Significance was established at *p* < 0.05.

**Results** After a mean 27-month follow-up, according to the MRI criteria, 60% of the women were cured in the VSF-AM group versus 45% in ASC group ( *p* = 0.52). The POP-Q and objective cure rates by MRI were correlated in the anterior vaginal wall ( *p* = 0.007), but no correlation was found with the subjective cure. The eLASV was larger among the patients with surgical failure, and a cutoff of ≥ 33.5 mm ^3^ was associated with postoperative failure (area under the receiver operating characteristic curve [ROC]: 0.813; *p* = 0.002).

**Conclusion** Both surgeries for prolapse were similar regarding the objective variables (POP-Q measurements and MRI cure rates). Larger eLASV areas were associated with surgical failure.

## Resumo

**Objetivo** A ressonância magnética (RM) tem sido considerada uma outra ferramenta para uso pré e pós-operatório em casos de prolapso de órgãos pélvicos. Contudo, pouco consenso existe sobre a sua prática para prolapso e a associação entre as linhas de referência da RM e o exame físico. Nós objetivamos avaliar resultados de médio a longo prazo de duas técnicas cirúrgicas para prolapso apical.

**Métodos** Um total de 40 mulheres com prolapso apical foram submetidas entre 2014 a 2016 a sacrocolpopexia abdominal (grupo SCA; n = 20) ou fixação bilateral vaginal no ligamento sacroespinhoso com tela anterior (grupo FVLS-TA; n = 20). Os exames físicos com estadiamento usando o Pelvic Organ Prolapse Quantification System (POP-Q: cura objetiva), e o International Consultation on Incontinence Questionnaire-Vaginal Symptoms (ICIQ-VS: cura subjetiva) foram analisados antes e depois de um ano da cirurgia, respectivamente. O exame de RM (linha pubococcígea [LPC], base vesical [BV], junção anorretal [JAR] e o volume subtendido estimado do levantador do ânus [VSELA]) foi realizado um ano antes da cirurgia. Estabeleceu-se o nível de significância em 5%.

**Resultados** Depois de uma média de 27 meses de seguimento, de acordo com a RM, 60% das mulheres foram curadas no grupo FVLS-TA versus 45% no grupo SCA ( *p* = 0.52). As curas objetivas associadas ao POP-Q e à RM foram correlacionadas na parede vaginal anterior ( *p* = 0.007), mas nenhuma correlação foi encontrada com a cura subjetiva. O VSELA foi maior entre as pacientes com fracasso da cirurgia, e um ponto de corte de ≥ 33.5 mm ^3^ esteve associado ao fracasso da cirurgia (area sob a curva característica de operação do receptor [COR]: 0.813; *p* = 0.002).

**Conclusão** Ambas as cirurgias para o prolapso foram similares nas curas objetivas tanto pelo POP-Q quanto pela RM. Áreas maiores de VSELA estiveram associadas com o fracasso das cirurgias.

## Introduction

Pelvic organ prolapse (POP) is a major health issue worldwide, and treatment is often surgical. However, reoperation rates may vary from 5% to 40%; up to 30% of the patients undergo more than 1 surgery throughout their lives. 1 The symptoms of POP are not always associated with physical examination findings, and the role of magnetic resonance imaging (MRI) in cases of POP has been investigated in recent years. 2 3 4

Many lines of reference and different protocols are used for the evaluation, and the most frequently used is the pubococcygeal line (PCL). 5 This line appears to have the best interobserver correlation and accordance with POP quantification systems (such as the Pelvic Organ Prolapse Quantification System, POP-Q), especially in the vaginal anterior wall. 4 In 2017, the European Society of Urogenital Radiology (ESUR) and the European Society of Gastrointestinal and Abdominal Radiology (ESGAR) released a joint recommendation guideline for MRI in POP, also recommending the PCL as the line of reference. 6

The role of the levator ani muscle (LAM) complex in pelvic support has been thoroughly described in the literature, as LAM alterations occur in women with POP, and are described on both ultrasound and MRI. 7 Rodrigues Junior et al. 8 have recently described the estimated levator ani subtended volume (eLASV) through MRI, and have showed significant posterior enlargement in patients with surgical failure following reconstructive prolapse surgery. 8 9

Apical defects (uterine prolapse, vault prolapse) are usually corrected by abdominal sacrocolpopexy (ASC) or vaginal sacrospinous fixation (VSF). In a previous study (Juliato et al. 10 ), our group showed that using an anterior mesh (VSF-AM) in the anterior compartment, the vaginal axis is deviated in both surgeries, however with no difference between them. 10 Nonetheless, we still have little data about the behavior of the MRI lines of reference for patients operated for POP and their association with physical examination findings and subjective cure using standardized questionnaires. Thus, we aimed to study these postoperative findings. Moreover, a receiver operating characteristic (ROC) curve was built for eLASV to look for a cutoff point as a failure of the surgical treatment.

## Methods

### Study Design

A secondary analysis of a prospective, randomized, single-blinded, two-arm, parallel study was performed from 2016 to 2017 in the Pelvic Floor Dysfunction Division, Department of Obstetrics and Gynecology, Faculdade de Ciências Médicas, Universidade Estadual de Campinas (FCM/UNICAMP). The present study was approved by the institutional Review Board (CAAE 64652217.0.0000.5404), and this trial was registered at the Clinical Trials Registry ( http://www.ensaiosclinicos.gov.br , code RBR-7t6rg2). The present study followed the Consolidated Standards of Reporting Trials (CONSORT) guidelines.

### Inclusion Criteria

The randomized controlled trial (RCT) included postmenopausal women aged 55 to 75 years with advanced POP (stage 3 or 4 according to the POP-Q) with no current hormone replacement therapy, and no previous gynecological surgery or malignancy. 11 These women were randomized by a computer-generated sequence. The attending physicians in the postoperative period were blinded to the surgery performed. All surgeries were performed from 2014 to 2016 in the Women's Hospital at UNICAMP, and all women were revaluated at least one year postoperatively.

### Exclusion Criteria

The exclusion criteria were the use of metallic implants (intrauterine devices, cardiac valves or stents, aneurism clips, orthopedic implants, or piercings), use of electronic implants (cardiac pacemakers, cochlear implants), claustrophobia, or recent tattoos.

### Interventions

**Fig. 1:**
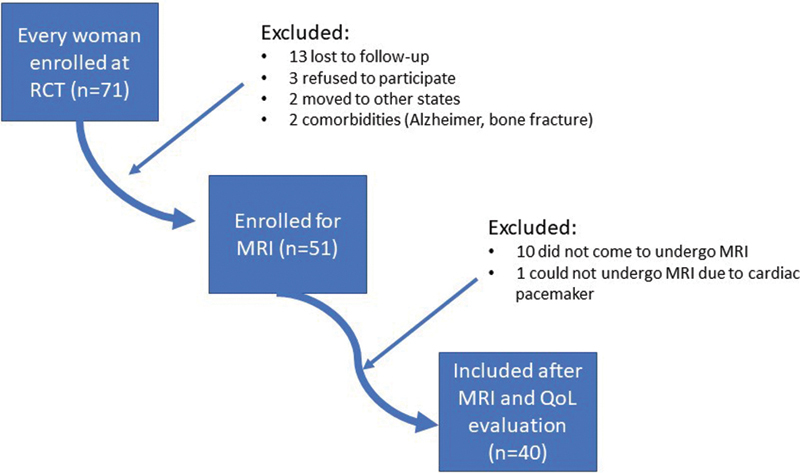
Flowchart depicting the recruitment process of the patients.

The women were randomized into two groups ( Fig. 1 ). The first group underwent surgery with hysterectomy and bilateral vaginal sacrospinous fixation with an anterior mesh (VSF-AM group). To correct the anterior vaginal wall, a polyvinylidene fluoride mesh (DynaMesh, FEG Textiltechnik, Aachen, North Rhine-Westphalia, Germany) was used in this group. The second group underwent abdominal supracervical hysterectomy with abdominal sacralcolpopexy (ASC group). Details of both intervention arms were published elsewhere. 11 If the patients presented concomitant stress urinary incontinence, they did not receive treatment for incontinence.

After at least one year of follow-up, the women underwent a physical examination performed by only one trained urogynecologist with at least five years of experience with the POP-Q who was blinded to the surgery performed. 12 During this visit, all women were instructed to answer a questionnaire regarding vaginal symptoms (International Consultation on Incontinence Questionnaire-Vaginal Symptoms, ICIQ-VS). 13 The MRI was scheduled no more than two weeks after the physical examination.

### MRI Protocol

Thei MRI was performed postoperatively. The women were instructed to empty their bladder within 2 hours before the MRI, and were taught the Valsalva maneuver for at least 20 seconds by a senior urogynecologist. Images were acquired in the supine position at rest and at maximum Valsalva using a 1.5-Tesla Philips Achieva Scanner v3.2 (Philips Medical Systems, Best, The Netherlands) with a 4-channel phased-array coil. At rest, T1-weighted sequences were obtained in the sagittal, coronal, and axial planes for the estimation of the pelvic anatomical structure and image planning. After vaginal introduction of 30 mL of ultrasound gel for better evaluation of the contours, T2-weighted single-shot fast spin-echo dynamic rest and Valsalva images were obtained through the midsagittal and parasagittal pelvic planes using the following parameters: repetition time, 1,300 milliseconds; echo time, 60 milliseconds; slice thickness, 6 mm; field of view, 36 cm; matrix size, 256 × 160. The description of this technique did not include one step of the ESUR/ESGAR guidelines with regard to the use of ultrasound gel in the rectal canal; we have had limitations regarding the use of this material in the steps to analyze rectal function (rest, straining, squeezing and evacuation). The images were interpreted with the aid of the MicroDicom software (Medical Imaging & Technology Alliance, Arlington, VA, US), using the PCL as references. 6 All images were analyzed independently by two professionals: a radiologist (SSD) with 18 years of experience in pelvic floor imaging, and a urogynecologist (LCSJ) with 5 years of experience in pelvic imaging. Both physicians were blinded to the surgery performed in each patient.

### Outcomes

**Fig. 2:**
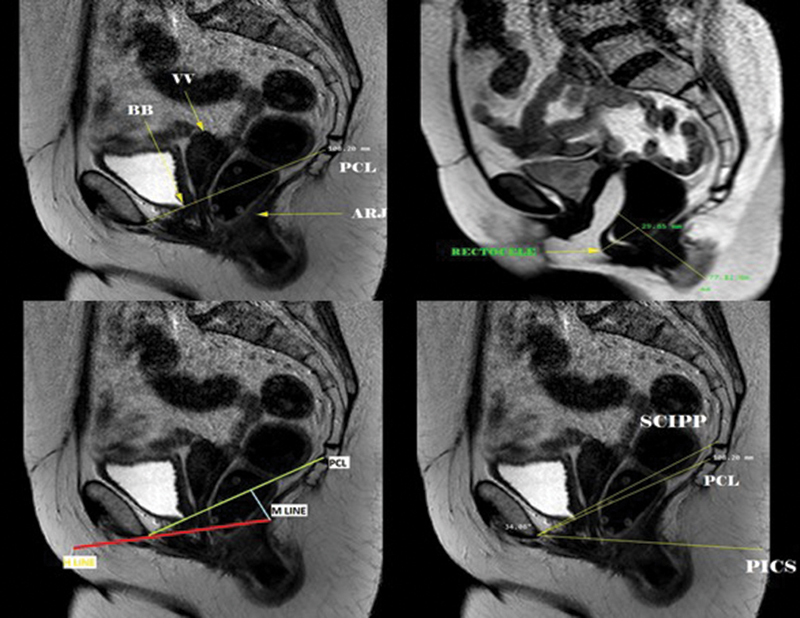
MRI in sagittal view depicting anatomical points and lines of reference (static and dynamic). Upper left (static); abbreviations: ARJ, anorectal junction; BB, bladder base; PCL, pubococcygeal line; VV, vaginal vault. Upper right (dynamic): rectocele pouch measure. Bottom left (static); red: H-line; light blue: M-line; yellow: pubococcygeal line. Bottom right (static); abbreviations: PCL, pubococcygeal line; PICS, pelvic inclination correction system line; SCIPP, sacrococcygeal inferior pubic point line.

The primary endpoint was the postoperative mid- and long-term MRI findings for both surgical techniques. The anatomical MRI references followed the ESUR/ESGAR recommendations ( Fig. 2 ). 6

The staging of the POP followed the ESUR/ESGAR recommendations. Regarding the PCL, the “rule of three” was used for the anterior and apical walls, and the POP was considered pathological starting at grade 2. 14 The anorectal junction (ARJ) has a different staging system; an ARJ descent > 5 cm is defined as grade II. 15 Measurement of the rectocele followed the “rule of two,” with grade II (> 4 cm) considered pathological. 7 16

Failure of the MRI was defined as bladder base stage ≥ 2, vaginal vault stage ≥ 2, or rectocele pouch stage ≥ 2. 6 Objective failure was defined as any point of prolapse beyond the hymen in the POP-Q (Points Ba [point B in the anterior vaginal wall, in centimeters]; Bp [point B in the posterior wall, in centimeters]; or C [point C in the apical wall] > 0). 17 Subjective cure was considered when ICIQ-VS questions 5a and 6a were equal to zero.

The H line (anteroposterior width of the levator hiatus), M-line (represent distance of its descent) and the width of the levator hiatus were also measured in millimeters in static MRI to calculate the eLASV: eLASV in mm ^3^ = −72.838 + 0.598 (H-line) + 1.217 (M-line) + 1.136 (width of levator hiatus). 9

The variables of the physical examination were basically defined by POP-Q measurements. 12 Sociodemographic characteristics were also recorded.

### Statistical Analysis

Objective measures, subjective measures, and cure rates are expressed as means or percentages, and the groups were compared using the Chi-squared test and the Fisher exact test. The Fisher exact test was used to compare the objective cure rate by physical examination, subjective cure rate, and MRI-based cure. The MRI and POP-Q were compared using the PCL as a line of reference. We also calculated the eLASV for each patient and compared it with surgical failure. A ROC curve was calculated to look for a cut-off point so that the eLASV could predict worse surgical outcomes. Correlations between the different methods (MRI versus POP-Q) were calculated using the Spearman correlation index. Values of *p* < 0.05 were considered statistically significant. The data were analyzed using the Statistical Analysis System (SAS, SAS Institute, Cary, NC, US) software for Windows, version 9.2.

## Results

### MRI and Physical Examination Results

Fig. 1 shows the flowchart of the studied patients, and Table 1 displays their sociodemographic characteristics. Out of 71 women from the original RCT who were screened for the present study, 12 could not be reached (lost to follow-up), and another 19 patients dropped out because of: refusal to participate ( *n* = 3), moved to other states ( *n* = 3), health restrictions ( *n* = 2), MRI restrictions (cardiac pacemaker; *n* = 1), and absence from 2 scheduled MRI examinations ( *n* = 10) ( Fig. 1 ). The final sample comprised 40 women. The mean follow-up was of 27 months. Table 1 shows the baseline characteristics and both groups are similar, except for a higher mean age of menopause for the ASC group ( *p* = 0.009).

**Table 1:** Sample demographics and preoperative status

Variables	ASC ( *n* = 20)	VSF-AM ( *n* = 20)	*p* -value
**Age (years) – mean (±SD)**	67.9 (4.9)	67.1 (4.82)	0.75
** < 60 years**	1	2	
** 60–69 years**	12	10	
** > 70 years**	7	8	
**Pregnancies – mean (±SD)**	4.85 (3.7)	5.4 (3)	0.28
**Vaginal delivery – mean (±SD)**	4.15 (3.39)	4.35 (3.08)	0.74
** Body mass index (kg/m ^2^ ) – mean (±SD) **	27.06 (3.72)	25.79 (4.84)	0.26
**Tobacco use (current or previous)**	3	4	0.66
**Menopause (years of age) – mean (±SD)**	51.85 (3.67)	48.4 (4.25)	0.009
**Preoperative POP-Q**			
**Anterior**			1.0
**stages → 2**	1	0	
** 3**	10	10	
** 4**	9	10	
**Apical**			0.75
**stages → 3**	11	10	
**4**	9	10	
**Posterior**			0.79
**stages → 0**	1	2	
** 1**	31	2	
** 2**	6	3	
** 3**	3	3	
** 4**	9	10	
**GH – mean, in centimeters (±SD)**	3.5 (1.08)	3.37 (1.72)	0.06
**PB – mean, in centimeters (±SD)**	2.15 (0.72)	2.05 (0.77)	0.77

Abbreviations: ASC, abdominal sacrocolpopexy; GH, genital hiatus; PB, perineal body; POP, Pelvic Organ Prolapse; POP-Q, Pelvic Organ Prolapse Quantification System; SD, standard deviation; VSF-AM, vaginal sacrospinous fixation with an anterior mesh.

Regarding the MRI results, the mean kappa coefficient between the two professionals presented good reliability (0.7). The measurements of the vaginal vault were larger for the VSF-AM group than for the ASC group (−0.08 ± 1.84 cm versus −1.43 ± 1.07 cm respectively; *p* = 0.007). The mean rectocele pouch was significantly larger in the ASC than in the VSF-AM group (3.91 ± 0.98 cm versus 3.38 ± 0.66 cm respectively; *p* = 0.033). The MRI staging showed no significant difference between the groups with regard to the anterior wall ( *p* = 0.73), the apical segment ( *p* = 1.0), or the posterior wall (ARJ) ( *p* = 1.0); however, the rectocele staging revealed significantly more stage-2 diagnoses in the ASC group ( *p* = 0.013) ( Table 2 ).

**Table 2:** MRI and POP-Q measurements of the operated patients

Variables	VSF-M ( *n* = 20)	ASC ( *n* = 20)	*p* -value
**Objective cure**	13	15	0.49
**Subjective cure**	17	17	1.0
**MRI measures**			
**Bladder base – mean (±SD)**	1.6 (1.8)	1.1 (1.6)	0.51
**Vaginal vault – mean (±SD)**	-0.08 (1.8)	-1.4 (1.0)	**0.007**
**Anorectal junction – mean (±SD)**	2.4 (0.9)	2.5 (1.0)	0.93
**Rectocele – mean (±SD)**	3.3 (0.6)	3.9 (0.9)	**0.033**
**Bladder base MRI stage**			
** Stages 0 and 1**	13	14	0.73
** Stage ≥2**	7	6	0.73
**Vaginal vault MRI stage**			
** Stages 0 and 1**	16	17	1.0
** Stage ≥2**	4	3	1.0
**Anorectal junction MRI stage**			
** Stage 0**	14	14	1.0
** Stage 1 or 2**	6	6	1.0
**Rectocele MRI stage**			
** Stage 0 or 1**	18	11	
** Stage 2**	2	9	0.0013
**MRI cure**	12	9	0.52
**Apical POP-Q**			
** stage 0**	12	14	0.74
** stage 1**	7	6	
** stage 2**	1	0	
**Anterior (bladder)**			
** stage 0**	7	8	0.27
** stage 1**	2	2	
** stage 2**	7	10	
** stage 3**	4	0	
**Posterior (rectal)**			
** stage 0**	19	11	0.013
** stage 1**	0	4	
** stage 2**	1	4	
** stage 3**	0	1	

Abbreviations: ASC, abdominal sacrocolpopexy; MRI, Magnetic resonance imaging; POP-Q, pelvic organ prolapse quantification; SD, standard deviation; VSF-AM, vaginal sacrospinous fixation with an anterior mesh.

When the physical examination using the POP-Q was performed, more patients in the ASC group had stage 2 and 3 posterior prolapses than those in the VSF-AM group (5 versus 1 respectively; *p* = 0.013). The rate of objective cure was of 65% ( *n* = 13) and 75% ( *n* = 15) in the VSF-AM and ASC groups respectively ( *p* = 0.49) ( Table 2 ). The rate of subjective cure did not present any difference between the groups; in total 85% of the women were cured according the ICIQ-VS ( *p* = 1.0) ( Table 2 ).

### MRI versus Physical Examination – POP-Q

When comparing failure rates between both methods (MRI and physical examination), there was a significant correlation with anterior wall failure: 13 patients had MRI-based failures, and 11 had POP-Q failures ( *p* = 0.002) and general failures ( *p* = 0.007). The MRI detected more apical failures than physical examination (7 versus 0). In the posterior segment, there were only 2 POP-Q failures versus 12 MRI-based failures with regard to the ARJ ( *p* = 0.08), and 11 failures after rectocele pouch MRI-staging ( *p* = 0.47). Using subjective failure as a reference, we found no association between the POP-Q findings and the rate of subjective cure ( *p* = 0.55); we have also found no association between POP-Q measurements and MRI-based cure in general ( *p* = 0.6), in the anterior wall (bladder base; *p* = 1.0), in the apical wall (vaginal vault; *p* = 1.0), or in the posterior wall (rectocele; *p* = 0.12) ( Table 3 ).

**Table 3:** Comparison among MRI, physical examination (POP-Q) and subjective cure rates

Vaginal wall - n (%)	MRI failure	POP-Q Failure	*p* -value	Subjective failure	*p* -value
**General**	19 (47.5)	12 (30)	0.007 a	6 (15)	0.6 e 0.055 f
**Anterior**	13 (32.5)	11 (27.5)	0.02 b	6 (15)	0.31 g 1.0 h
**Apical**	7 (17.5)	0	*	6 (15)	*
**Posterior**					
**ARJ**	12 (30)	2 (5)	0.08 c	6 (15)	0.6 i
**Rectocele**	11 (2.5)	2 (5)	0.47 d		0.12 j

Abbreviations: ARJ, anorectal junction; MRI, magnetic resonance imaging; POP-Q, Pelvic Organ Prolapse Quantification System.

Notes:

a MRI general failure versus POP-Q general failure (Fisher exact test).

b Bladder base to PCL in the MRI (grade ≥ 2) versus POP-Q Ba > 0 (Fisher exact test).

c Anorectal junction to PCL in the MRI (grade ≥ 1) versus POP-Q Bp > 0.

d Rectocele measure in the MRI (grade ≥ 2) versus POP-Q Bp > 0.

e Subjective cure in the ICIQ-VS versus general MRI cure.

f Subjective cure in the ICIQ-VS versus POP-Q general failure.

g Subjective cure in the ICIQ-VS versus POP-Q Ba > 0 (grade ≥ 2).

h Subjective cure in the ICIQ-VS versus BB to PCL in the MRI.

i Subjective cure in the ICIQ-VS versus ARJ to PCL in the MRI (grade ≥ 1).

j Subjective cure in the ICIQ-VS versus rectocele pouch stage in the MRI (grade ≥ 2).

* Vaginal vault to PCL in the MRI (stage ≥ 2) versus POP-Q C > 0 and subjective cure (ICIQ-VS questions 5a and 6a equal to zero) – homogenous sample, no test was made.

Abbreviations: ARJ, anorectal juntion; Ba, point B in the anterior vaginal wall, in centimeters;BB, bladder base; Bp, point B in the posterior wall, in centimeters; C, point C in the apical wall, in centimeters; ICIQ-VS, international consultation on incontinence questionnaire - vaginal symptoms; MRI, Magnetic resonance imaging; PCL, pubococcygeal line; POP-Q, pelvic organ prolapse quantification.

### Levator Ani Subtended Volume (LASV)

The width of the levator hiatus and the length of the H- and M-lines were measured in static MRI for eLASV calculation, and the numbers were compared by dividing the sample according to POP-Q success. The H- and M-lines were significantly longer among the patients who presented POP-Q failure than among those who achieved objective success (69.6 ± 5.6 mm versus 62.2 ± 7.8 mm respectively; *p* = 0.004; and 23.5 ± 7.8 mm versus 16.7 ± 6.9 mm respectively; *p* = 0.004). The width of the levator hiatus was similar: 38.5 ± 8.1 mm for the patients with failure versus 34.6 ± 5.5 mm for those with success ( *p* = 0.08).

**Fig. 3:**
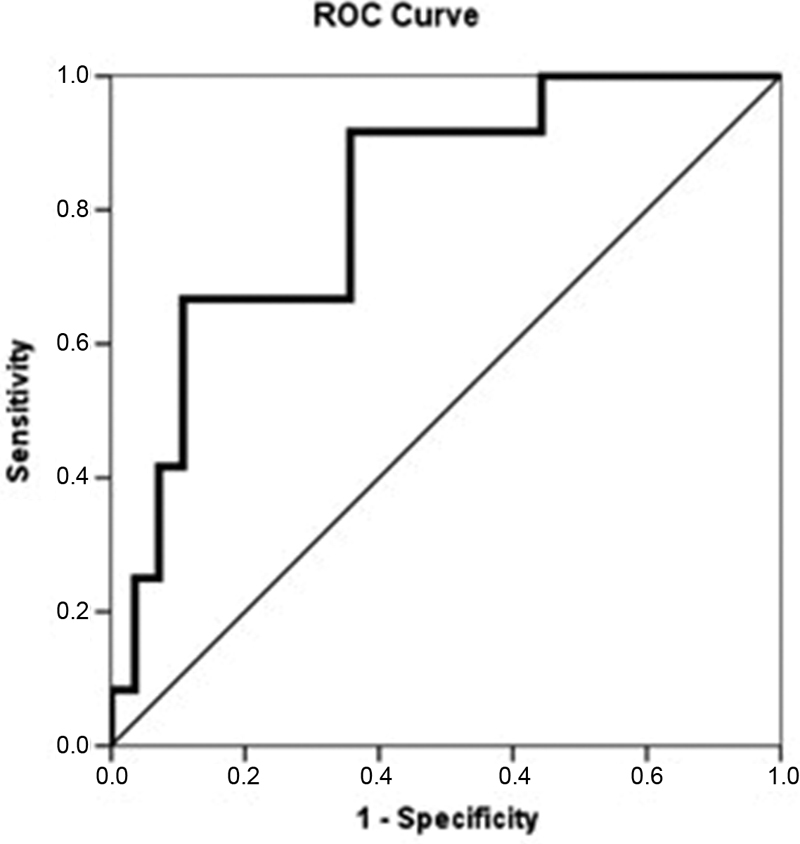
eLASV measurement (ROC curve). Area under the curve: 0.813; *p* = 0.002; 95%CI: 0.672–0.953; cutoff point: eLASV ≥ 33.5 mm3.

The eLASV was calculated for each patient, and it was larger among those with failure than among those with success (41.1 ± 16.5 mm ^3^ versus 24.1 ± 13.6 mm ^3^ respectively; *p* = 0.002). The area under the ROC curve was of 0.813 ( *p* = 0.002; confidence interval [CI] = 0.672–0.953), and the optimal cutoff was of 33.5 mm ^3^ , with specificity of 89.2% (70.6% to 97.1%), sensitivity of 66.6% (35.4% to 88.7%), and accuracy of 83.5% (66.4% to 92.1%). Among the patients who experienced surgical failure, 66.6% had an eLASV of ≥ 33.5 mm ^3^ versus 10.7% among those who experienced surgical success ( *p* < 0.001) ( Fig. 3 ).

## Discussion

The present study showed good results for both vaginal and abdominal surgeries, especially regarding subjective outcomes at the mean 27-month follow-up. The MRI staging showed greater failures compared with the POP-Q, and neither correlated well with the symptomatology.

In their nonrandomized study, Ginath et al. 18 compared two surgical approaches (ASC versus Prolift vaginal mesh) and showed equally good postoperative results for both techniques in the POP-Q, but not in the MRI, which revealed no difference between the preoperative and postoperative anatomical positions and angles relative to the PCL. Van der Weiden et al. 19 evaluated 43 women at 6 months after laparoscopic ASC, and found great improvement in prolapse measures for all compartments, but only for the apex in the MRI assessment, resulting in a poor correlation with the POP-Q. 19 Conversely, Brocker et al. 20 studied 69 patients who underwent vaginal mesh repair for POP; during the one-year follow-up, both the dynamic MRI and the clinical examination showed good results, but the MRI showed more prolapse.

The correlation between the POP-Q and the symptoms is frequently poor because the symptoms do not necessarily correlate with the severity of the POP. 21 The MRI assessment seems to be even more dissociated from the symptomatology – MRI scans often describe a great number of prolapses in asymptomatic women. 22 We also found no significant correlation between subjective cure or quality of life questionnaire scores and MRI-based prolapse staging and failure definitions.

Several studies 4 23 have compared physical examination findings with different lines of reference and points of anatomical interest in the MRI, mainly with poor results. The anterior wall seems to show good accordance. 2 Our results were also better for the anterior compartment in terms of a significant association between MRI-based pathological POP and a Ba point > 0 in the POP-Q. We found a rate of 70% of objective cure, a rate of 85% of subjective cure, and a rate of 52.5% of MRI-based cure for the whole sample, without a significant association among symptomatology, POP-Q, and MRI. The MRI staging system showed a greater number of pathological prolapses than the POP-Q and the symptoms in our sample.

The MRI has become an important asset in the evaluation of pelvic-floor dysfunction. 24 The most commonly used line of reference, the PCL, is also the one with the greatest interobserver correlation indexes. 7 This line unites the pubic bone and coccyx, but precise definitions vary among studies. We used the PCL as described by El Sayed et al. 6 in 2017,; that is, a line from the inferior tip of the pubic bone to the anterior aspect of the last visible coccygeal joint.

Another important contribution of the MRI is the proper evaluation of the eLASV and its role in pelvic support. We intended to do a study similar to the research by Wyman et al. 9 ; they found a cutoff in the eLASV that correlated with surgical failure: they retrospectively analyzed a cohort of 66 women who underwent laparoscopic uterosacral ligament suspension, and found a significant association between the eLASV and surgical failure at the one-year follow-up, with a cutoff value of 38.5 mm ^3^ . In our study, an eLASV ≥ 33.5 mm ^3^ was associated with surgical failure, with good accuracy. However, as this parameter was analyzed postoperatively, we cannot say that this would be a predictor of surgical failure, but as a diagnosis and/or audit of the surgery performed. Our sample might have comprised women with worse preoperative prolapses, and a smaller eLASV could be associated with failure, given that other important factors such as age or parity also play important roles in surgical outcomes. Apart from that, we evaluated a different surgical modality.

Our study has many strengths. We studied a homogeneous group of women without previous surgical treatment who were randomized according to two surgical POP correction techniques for a mean follow-up  > 2 years. We have followed the latest recommendations regarding MRIs for prolapse. Our limitations are: we did not use rectal gel or perform MRI defecography, and this might have impaired our evaluation of the posterior wall. Additionally, almost all operations were performed for advanced cases of prolapse, which affected the preoperative MRI examinations of these patients for purposes of comparison. The assessment of the eLASV by postoperative MRI could have produced some bias, and the fact that the ARJ was not adequately visualized may have impaired the measurement of the H-line; this can alter the eLASV assessment as well. However, we do not believe that our surgical techniques directly affected the measurements used for this calculation (that is, the length of the H- and M-lines and the width of the levator hiatus). Finally, we included a limited number of patients, and further studies with larger samples are necessary.

## Conclusion

The MRI and POP-Q cure rates were associated, but only in the anterior wall. The MRI staging showed greater failures compared with the POP-Q, and neither correlated well with the symptomatology. Both surgical techniques for advanced prolapse were similar in terms of the objective POP-Q and MRI cure rates. The objective results were poorer in the posterior compartment in the ASC group. The eLASV was larger among the patients whho experienced surgical failure, and when this value was ≥ 33.5 mm ^3^ , there was a significant increase in the chance of postoperative failure, but with the limitation that it was only analyzed postoperatively.
